# Egyptian adaptation and validation of the Edinburgh Cognitive and Behavioral Amyotrophic Lateral Sclerosis Screen (ECAS*-EG*)

**DOI:** 10.1007/s10072-023-06639-6

**Published:** 2023-02-08

**Authors:** Radwa Soliman, Hebatallah R. Rashed, Ramez R. Moustafa, Nabila Hamdi, Mahmoud S. Swelam, Ahmad Osman, Nagia Fahmy

**Affiliations:** 1 Neuromuscular Unit, Neurology and Psychiatry Department, Faculty of Medicine, Ain Shams University, Cairo, 11566 Egypt; 2 Molecular Pathology Unit, German University in Cairo (GUC), Cairo, Egypt; 3 Biotechnology Department, Basic and Applied Sciences Institute, Egypt-Japan University of Science and Technology, Borg Al Arab, 21934 Egypt; 4 Biochemistry Department, Faculty of Science, Ain Shams University, Cairo, 11566 Egypt

**Keywords:** Amyotrophic lateral sclerosis, ALS, ECAS, Cognitive, ALSFTD

## Abstract

**Background:**

Amyotrophic lateral sclerosis (ALS) is the most common, fatal adult neuromuscular disease. It is a multi-system disorder characterized primarily by motor manifestations, but there is established evidence for cognitive and behavioral impairment, which is associated with poor prognosis, hence, the importance of tools for its assessment. The Edinburgh Cognitive and Behavioral Assessment Screen (ECAS) is an invaluable assessment tool for cognition in ALS-front temporal spectrum dementia (FTSD), as it accommodates physical challenges that usually confound traditional neuropsychological testing in those patients.

**Objective and methods:**

To validate the Egyptian Arabic version of ECAS (ECAS-EG) based on the original English scale. This is a prospective study. The ECAS was adapted and administered to 62 Egyptian ALS patients and 60 healthy controls. Patients were recruited from the Neuromuscular Unit, Ain Shams University Hospital. The ECAS was adapted to Egyptian Arabic after being translated using the back translation method. Internal consistency of the test, inter-rater reliability, and construct validity were assessed.

**Results:**

The Egyptian Arabic version of ECAS (ECAS-EG) showed good internal consistency using Cronbach’s alpha of 0.84. Inter-rater reliability was tested, values for all variables were compared, and no statistically significant differences were found (ICC = .997). ECAS-EG discriminated significantly between the patients from the control subjects (*p-value* of 0.001). There was a strong positive correlation between the ECAS-EG total score and the MoCA total score with a *p-value* of 0.001, thus indicating convergent validity. The test showed that 63% of Egyptian ALS patients were cognitively affected; most affected domains were executive functions and verbal fluency.

**Conclusion:**

The current study proves that the Egyptian version of the ECAS (ECAS-EG) is valid and reliable among Egyptian ALS patients and it would be applicable to the general Arabic-speaking population.

**Supplementary information:**

The online version contains supplementary material available at 10.1007/s10072-023-06639-6.

## Introduction

Amyotrophic lateral sclerosis (ALS) is one of the most common fatal motor neuron disorders. Recent studies have shown that the incidence of ALS varies between 0.6 and 3.8 per 100,000 using the person-year calculation method, while the prevalence varies between 4.1 and 8.4/100,000 [1–4].

Despite the increased awareness of ALS as a multi-system disorder, characterized primarily by a progressive lower motor neuron (LMN) and upper motor neuron (UMN) degeneration, there is established evidence for non-motor symptoms in ALS, which are now recognized as a prominent integral feature of the disease [5, 6].

Among the most important non-motor symptoms of ALS are cognitive and behavioral changes. Cognitive dysfunction typically encountered in ALS includes deficiencies in frontal executive skills, varying from mild deficits to definite frontotemporal dementia (FTD) [7].

A significant overlap between ALS and the behavioral variant of FTD (bvFTD) has been observed at clinical, genetic, and pathologic levels [8].

Associated cognitive impairment in ALS patients is a well-known negative prognostic factor contributing to functional decline and reduced survival; therefore, a better definition and assessment of this impairment is clearly relevant [9, 10].

The Edinburgh Cognitive and Behavioral Assessment Screen (ECAS) is an established, brief, multi-domain neuropsychological assessment which provides an effective measure of cognitive and behavior assessment of ALS. It is designed to accommodate physical disability commonly featured in ALS, especially in the advanced stages of the disease, thus allowing for both written and spoken responses. The assessment determines not only the presence of cognitive impairment but also its severity and nature; therefore, it is recommended as one of the principal tools for assessing cognition in ALS-FTSD [11].

ECAS assesses the following domains: executive functions, verbal fluency, and language (ALS-specific), along with memory and visuospatial abilities (non-ALS-specific). ECAS’s total score ranges from 0 (worst performance) to 136 (best performance). Moreover, a brief caregiver interview provides an assessment of behavior changes and psychotic symptoms usually associated with ALS patients.

Many adaptations for the ECAS have been carried out, such as the German-Swiss [11], Italian [12], Spanish [13], Norway [14], Chinese [15], and Czech [16]. Moreover, it has been validated in bvFTD without ALS and Alzheimer’s dementia [17]. Tunisian ECAS-AR was recently published [18].

## Objective

The aim of this study is to assess the cognitive status in a cohort of Egyptian ALS patients and validate the ECAS in Egyptian Arabic based on the original English version.

## Methods

### Patient’s recruitment

ALS patients were recruited consecutively from the Neuromuscular Unit, Ain Shams University hospitals, in the period between January 1st, 2019, and December 1st, 2021.

### Inclusions and exclusions

All patients presented to the neuromuscular clinic with definite ALS according to El Escorial revised criteria [19] were included. We excluded patients with severe respiratory dysfunction and illiterate patients who had not received any formal education.

### Healthy controls

Our study included healthy controls with no medical or family history of neurodegenerative diseases or psychiatric diseases with matched age, sex, and level of education.

### Ethical consideration

Our study was approved by the ethical committee and institutional review board of the faculty of medicine, Ain Shams University, Cairo, Egypt, in accordance with the Declaration of Helsinki (WMA, 1964). Approval was obtained from the ECAS’ copyright holder, Dr. Sharon Abrahams, to validate the test in Arabic. All participants signed consent for participation.

### ECAS adaptation

The study was performed in two stages. In the first stage, the original scale was translated including the caregiver interview for behavioral domains into Egyptian Arabic using the back translation method. In the second stage, we applied the Egyptian version of the scale.

A few points were modified in the test so that it would be more compatible with the Egyptian participants and also to guarantee its efficacy considering the individuality of the Arabic language such as follows.

1. The language spelling category: we changed four words (highlighted in Table 1) so that their Arabic translation would have wider variability as regards the word’s length and difficulty of their spelling, but still, we were committed to the ECAS guidelines instructions of having 4 nouns, 4 verbs, and 4 compound words in this category (Table 1).
2. The verbal fluency: we developed the verbal fluency index for two Arabic letters **س**, **ك** (Table 2) after testing and comparing several Arabic letters on healthy controls using this equation:
  $$\mathrm{VFI}=\frac{\left(\mathrm{test}\;\mathrm{time}-\mathrm{time}\;\mathrm{taken}\;\mathrm{to}\;\mathrm{repeat}\;\mathrm{words}\right)}{\mathrm{number}\;\mathrm{of}\;\mathrm{correct}\;\mathrm{words}\;\mathrm{generated}}$$
3. Memory-immediate recall: Done a few modifications to the short story to make it more familiar, as we used Arabic names instead, as well as the name of the event and location. Words changed in the ECAS-EG and their back translation are highlighted in Table 3.

**Table 1:** Spelling category modifications

Original spelling test	Egyptian Arabic translation	Back translation
Envelope	ظرف	Envelope
Skateboard	فتح	Opened
Construction	معمار	Construction
Partner	شريك	Partner
Biscuit	كمبيوتر	Computer
Lawnmower	مصابيح	Lamps
Deliver	يوصل	Deliver
Recorded	يسجل	Record
Coat hanger	شماعة	Coat hanger
Orchestra	اوركسترا	Orchestra
Screwdriver	مهرجان	Festival
Brought	يتقصى	Investigate

**Table 2:** Conversion table to convert VFI to a fluency score for letters "سين"&" كا"

Score	Spoken VFI سين	Written VFI سين	Spoken VFI كاف	Written VFI كاف
0	>6.63	>2.76	>4.26	>22.03
2	5.39 to <6.62	2.75 to <2.25	3.46 to <4.25	17.81 to <22.02
4	4.15 to <5.38	2.24 to <1.74	3.45 to <2.66	13.60 to <17.80
6	2.91 to <4.14	1.73 to <1.22	1.86 to <2.65	9.39 to <13.59
8	1.68 to <2.90	1.21 to <0.71	1.06 to <1.85	5.17 to <9.38
10	0.44 to <1.67	0.70 to <0.199	0.26 to <1.05	0.96 to <5.16
12	<0.43	<0.198	<0.25	<0.95

*VFI*, verbal fluency index

**Table 3:** Immediate recall modifications

Original immediate recall	Egyptian Arabic translation	Back translation
**Last Sunday**, the **annual litter collection** took place in **Primrose Woods. Forty two** people joined in to remove old bicycles and shopping trolleys. **Mr Douglas Watt** from the woodland project told local reporters that he was very impressed and **especially proud** of **the 17 children** who came along	يوم **الأحد** الماضي، عُقد **المعرض السنوي لزهور الربيع** في **حديقة الأورمان**. حيث شارك فيها **أثنان و اربعون** شخص، **لعرض والاعتناء بالنباتات** .وقد صرح السيد/ **طارق وجدى** – من **وزارة الزراعة** - للمرارسلين المحليين بأنه كان **مبهورًا وفخورا بالأخص بالسبعة عشر طفلا** ، الذين حضروا للمشاركة.	Last **Sunday**, the **annual exhibition for spring flowers** took place at **the Orman park**. **Forty two** people joined in **to display and take care of the plants**. Mr/ **Tarek Wagdy** (from **the ministry of agriculture**) told local reporters that he was **impressed and especially proud** of the **17 children** who came along.

The first stage was executed by two Egyptian physicians who are fluent in English. Then, the translations were compared, and a modified Egyptian Arabic version was obtained. Then, the scale was translated back to English by another bilingual physician who had never had any contact with this scale. This version was compared with the original English version, and the final ECAS-EG was obtained***.***

In the second stage, the Arabic version of the test was administered on the same day by two neurologists who were trained on the ECAS scale. They administered the test separately on the same day, the first neurologist at the beginning of the visit and the second in the end, in order to test the inter-rater reliability.

One week later, patients were administered The Montreal Cognitive Assessment (MoCA) which is a brief 30-question test that assesses cognitive impairment [20]. The Arabic MoCA was administered in 2009 after its Arabic translation and adaptation. It showed high sensitivity (92.3%) and good specificity (85.7%) [21].

Subsequently, ECAS-EG total score was compared to MoCA total score; the scorings for all questions were compared, and the validity analysis of the scale was assessed.

Furthermore, primary caregivers completed a questionnaire for five-domain characteristic behavioral changes of FTD and three psychotic questions.

### Statistical methods

Data analysis was done using SPSS 25 (IBM SPSS Statistics for Windows, Version 25.0, IBM Corp., Armonk, NY). Quantitative data were presented as means and standard deviations. Qualitative data were presented as counts and percentages. Cronbach’s alpha was used to measure internal consistency. The association between the ECAS and the MoCa was tested by means of Pearson’s correlation. Intra-class correlation coefficient (ICC) was used to measure inter-rater reliability. Receiver operating characteristic (ROC) analyses were adopted to test the ability of the ECAS to discriminate ALS patients from healthy controls and to derive cut-off values. A *P*-value equal to or less than 0.05 was considered statistically significant.

## Results

### Demographic and clinical properties

A total of 62 ALS patients and 60 age, sex, and education-matched healthy controls were included between January 1st, 2019, and December 1st, 2021. The ALS group was 46 males and 16 females with a male-to-female ratio of 3:1. Mean age for the patients was 45.57 ± 13.13 years, and the control was 46.30 ± 11.52 years. The mean years of schooling for patients was 5.6 ± 1.98, and the control was 5.7 ± 1.75 (Table 4).

**Table 4:** Demographic data and clinical characteristics of the ALS cohort

Data/group		ALS (*n* = 62)	Controls (*n* = 60)	*p*-value*
Age	Mean/SD	45.57 ± 13.12 (25.0–66.0)	46.30 ± 11.52 (28.0–68.0)	0.16
Age of onset	Mean/SD	41.90 ± 12.52 (41.90–12.52)		
Sex	Male	46 (74%)	45 (75.0%)	
	Female	16 (26%)	15 (25.0%)	
	Ratio M:F**	3:1	3:1	0.67
Years of schooling	Mean	5.6 ± 1.98	5.7 ± 1.75	0.689
ALSFRS	Mean/SD	34.13 (19.0–43.0)		
Type	Sporadic	46 (74.19%)		
	Familial	16 (25.8%)		
Onset symptom	Weakness	31 (50.0%)		
	Dysphagia	18 (29.03%)		
	Fasciculation	13 (20.97%)		
Site of onset	Bulbar	18 (29.032%)		
	Spinal	44 (70.96%)		
Clinical phenotype	Classical spinal	37 (59.67%)		
	Classical Bulbar	18 (29.03%)		
	Flail arm or dropped head	7 (11.3%)		

^*^Student’s *t*-test

^**^Chi-square test (FE: fisher extract)

*ALSFRS-R*, ALS functional rating scale-revised**-**Arabic version [22]

### Reliability of ECAS-EG

ECAS-EG showed good internal consistency of the Arabic ECAS using Cronbach’s alpha of 0.84 (Table 5).

**Table 5:** Internal consistency of ECAS-EG

a) Reliability statistics		
Cronbach’s alpha	*N* of items	
0.84	14	
b) Item statistics		
	Mean	SD
Naming	6.08	1.060
Comprehension	6.53	1.141
Immediate recall	8.90	1.251
Spelling	9.39	2.614
Fluency سين	7.97	2.247
Reverse digit span	4.40	2.004
Alternation	6.34	2.088
Fluency كاف	5.48	2.941
Dot counting	3.39	.856
Cube counting	2.82	.967
Number location	3.32	.536
Social cognition	10.74	1.941
Delayed recall	8.02	1.509
Delayed recognition	3.05	.664

### Inter-rater reliability

Intra-class correlation showed a mean change of 0.99 of the total score from the first to the second evaluation of ECAS with 95% CI = .997 (.991–.999). No statistically significant differences were found (Table 6).

**Table 6:** Test-retest reliability for different items

	Mean	SD	Inter-item correlation	ICC (95% CI)
Naming 1	6.07	1.21	.977	.976 (.930–.992)
Naming 2	6.00	1.24		
Comprehension 1	6.36	1.50	.989	.986 (.957–.995)
Comprehension 2	6.29	1.64		
Spelling 1	8.71	2.92	.991	.992 (.975–.997)
Spelling 2	8.71	2.95		
Language 1	21.14	5.20	.998	.997 (.992–.999)
Language 2	21.00	5.32		
Fluency S 1	8.29	2.58	.979	.979 (.938–.993)
Fluency S 2	8.43	2.62		
Fluency T 1	6.00	2.94	.983	.983 (.950–.994)
Fluency T 2	5.86	2.88		
Verbal fluency 1	14.29	5.25	.989	.990 (.969–.997)
Verbal fluency 2	14.29	5.20		
Alternation 1	5.21	2.26	.994	.992 (.976–.997)
Alternation 2	5.21	2.26		
Reverse digit span 1	3.93	2.16	.986	.984 (.953–.995)
Reverse digit span 2	3.93	2.16		
Sentence completion 1	10.00	2.15	1.00	1.00 (1.00–1.00)
Sentence completion 2	9.71	2.02		
Social cognition 1	10.29	2.02	.983	.983 (.950–.994)
Social cognition 2	10.50	1.56		
Executive 1	28.71	4.63	.996	.995 (.984–.998)
Executive 2	28.71	4.63		
ALS-specific 1	64.14	12.41	.997	.997 (.991–.999)
ALS-specific 2	64.00	12.61		
Dot counting 1	3.43	1.02	.965	.965 (.899–.989)
Dot counting 2	3.36	1.01		
Cube counting 1	2.57	.94	.960	.960 (.883–.987)
Cube counting 2	2.50	.94		
Number location 1	3.21	.70	1.00	1.00 (1.00–1.00)
Number location 2	3.21	.70		
Visuospatial 1	9.21	2.36	.988	.987 (.961–.996)
Visuospatial 2	9.07	2.30		
Immediate recall 1	9.14	1.46	.876	.876 (.646–.955)
Immediate recall 2	8.86	1.46		
Delayed recall 1	7.64	1.95	1.00	1.00 (1.00–1.00)
Delayed recall 2	7.64	1.95		
Delayed recognition 1	3.29	.73	1.00	1.00 (1.00–1.00)
Delayed recognition 2	3.29	.73		
Memory 1	20.07	3.52	.978	.976 (.930–.992)
Memory 2	19.79	3.42		
ALS nonspecific 1	29.14	5.65	.994	.993 (.980–.998)
ALS nonspecific 2	28.86	5.63		
Total score 1	93.29	17.11	.997	.997 (.991–.999)
Total score 2	92.86	17.19		

*ICC*, intra-class correlation

### Validity and diagnostic properties of ECAS-EG

The ECAS-EG was able to differentiate between ALS patients and controls. It showed a mean total score in ALS patients of 96.10 ± 14.05, and in control, it was 107.6 ± 11.9, while the ALS-specific score in ALS patients was 66.6 ± 10.6, and in control, it was 76.53 ± 9.19. There was a significant difference in both total and ALS-specific scores (*p-value* of 0.001), thus indicating case-control discrimination (Table 7).

**Table 7:** Case-control discrimination (differentiate between ALS patients and controls as regards total ECAS-EG score and ALS-specific items score only)

	ALS patients		Control		Student’s *t*-test	*P*-value
	Mean	SD	Mean	SD		
Total ECAS-EG score	96.10	14.05	107.60	11.99	4.44	**<0.001**
ALS-specific items	66.60	10.61	76.53	9.19	5.05	**<0.001**

Values in bold denotes the significance of *P *- value thus indicate the ability of the ECAS-EG to diffrentiate between patients and control

**Fig. 1:**
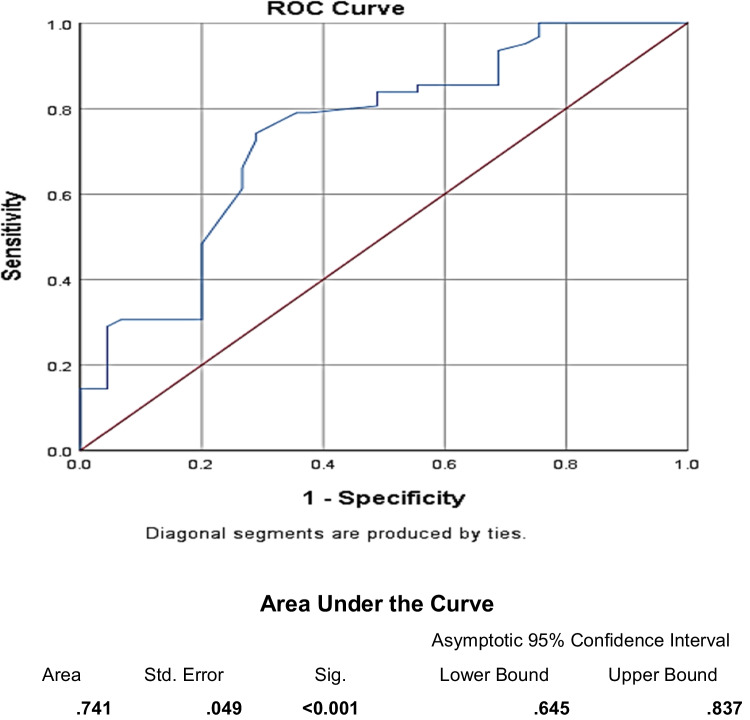
Receiver operating characteristic (ROC) curve for differentiation between ALS patients and controls according to ECAS-EG total score: best cut-off value ≤ 104, sensitivity = 79%, specificity = 64.4%

**Fig. 2:**
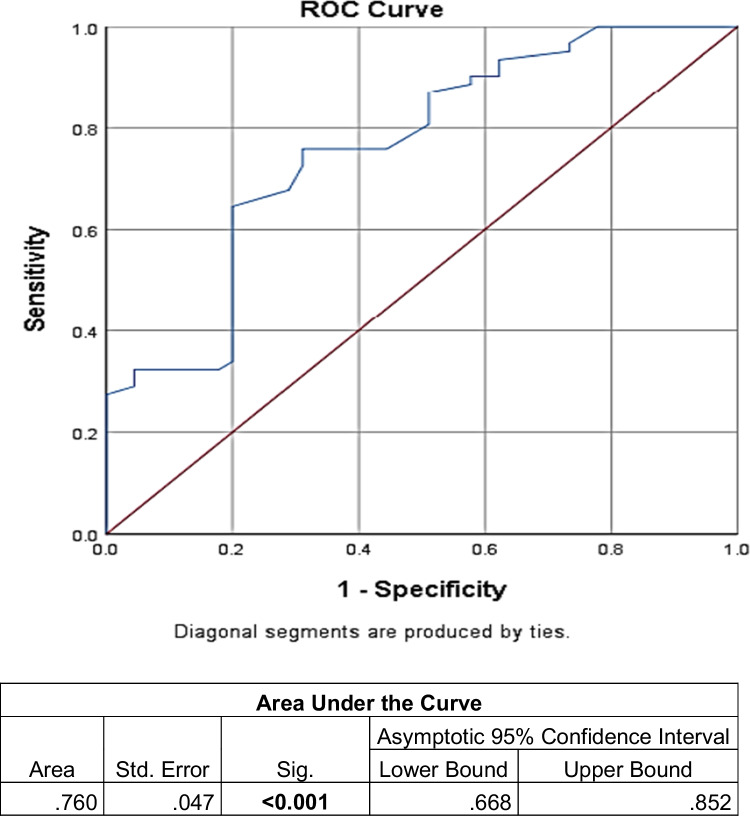
Receiver operating characteristic (ROC) curve for differentiation between ALS patients and controls according to ECAS-EG ALS-specific items score: best cut-off value ≤72, sensitivity = 72.6%, specificity = 68.9%

The ECAS-EG showed the best ALS cut-off value of ≤104, sensitivity = 79%, and specificity = 64.4% in total score (Fig. 1); 39 ALS patients (63%), according to ECAS, had cognitive impairment (scored less than 104). ALS-specific showed the best cut-off value of ≤72, sensitivity = 72.6%, and specificity = 68.9% (Fig. 2). Best cut-off value for each item is shown in Table 8.

**Table 8:** Best cut-off value for each ECAS-*EG* item

Test result variable(s)	Best cut-off value (≤)	Sensitivity	Specificity
Naming	6	73.2%	76.2%
Comprehension	6	51.2%	90.5%
Spelling	11	87.8%	42.9%
Language	22	56.1%	85.7%
Fluency س	9	80.5%	61.9%
Fluency ك	7	80.5%	52.4%
Verbal fluency	15	78.0%	61.9%
Alternation	8	95.1%	47.6%
Reverse digit span	5	43.9%	81.0%
Sentence completion	11	75.6%	57.1%
Social cognition	11	51.2%	71.4%
Executive	36	92.7%	19%
Dot counting	3	46.3%	71.4%
Cube counting	2	48.8%	85.7%
Number location	3	82.9%	71.4%
Visuospatial	9	53.7%	85.7%
Immediate recall	9	75.6%	95.2%
Delayed recall	8	73.2%	52.4%
Delayed recognition	3	82.9%	38.1%
Memory	21	80.5%	66.7%
ALS nonspecific	30	65.9%	81.0%

### Correlation between total ECAS and total MOCA score among ALS patients (convergent validity)

**Fig. 3:**
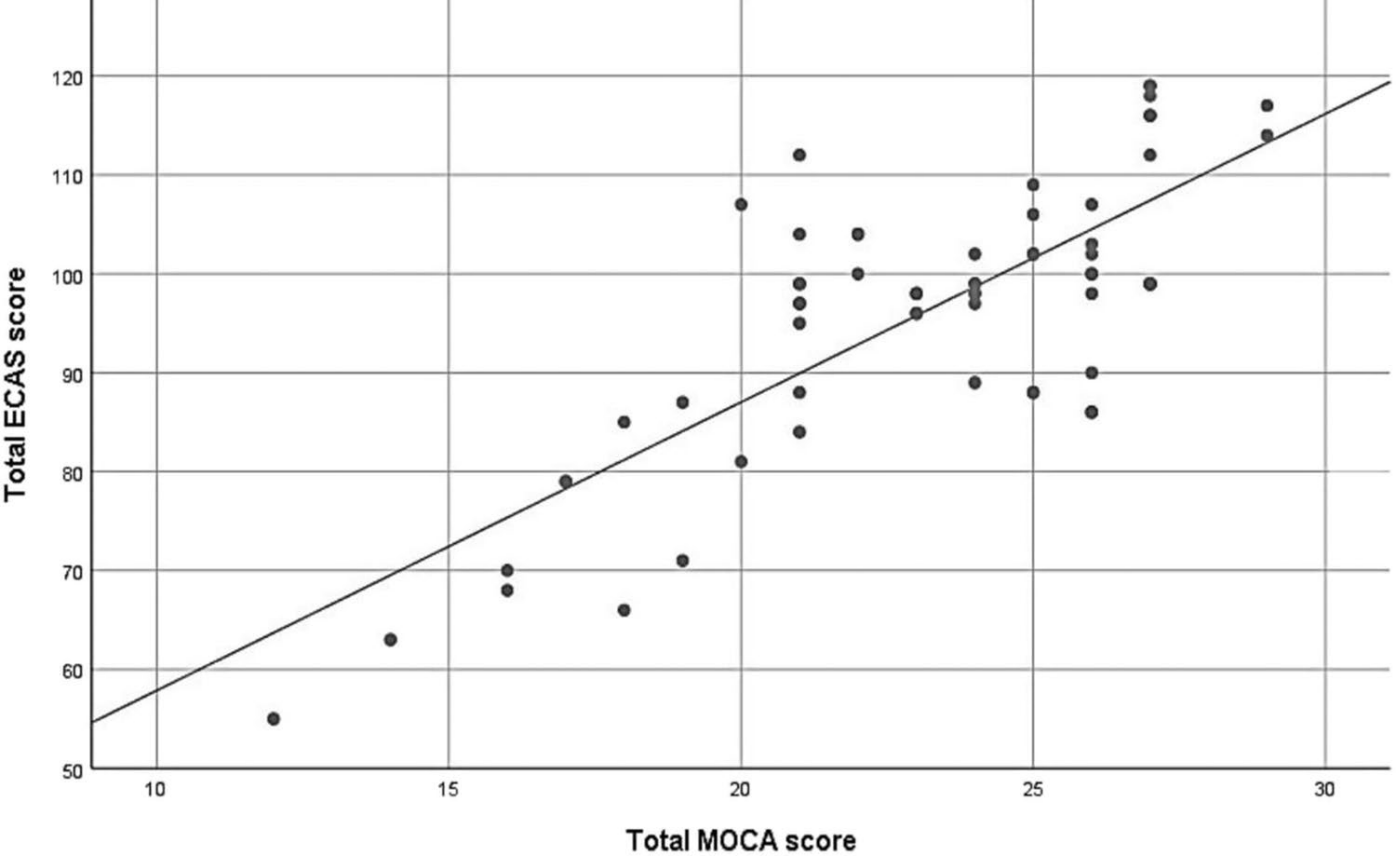
Scatter plot showing there is a strong positive correlation between ECAS-EG total score and MoCA total score, thus indicating convergent validity

The mean score of MOCA in the ALS group was 23.11 ± 3.74 (range: 12–29), denoting that 32 patients had cognitive impairment (scored less than 26 after adding one point for those with less than 12 years of education). We performed validity analysis in correlation to MOCA; there was a strong positive correlation between the ECAS-EG total score and the MoCA total score with a *p*-value of 0.001, thus indicating convergent validity (Table 9 and Fig. 3).

**Table 9:** Correlation between total ECAS-EG and total MOCA score among ALS patients

	Mean	SD	Pearson correlation	*P*-value
Total ECAS-*EG*	96.10	14.05	0.78	<0.001
Total MOCA	23.11	3.74

**Fig. 4:**
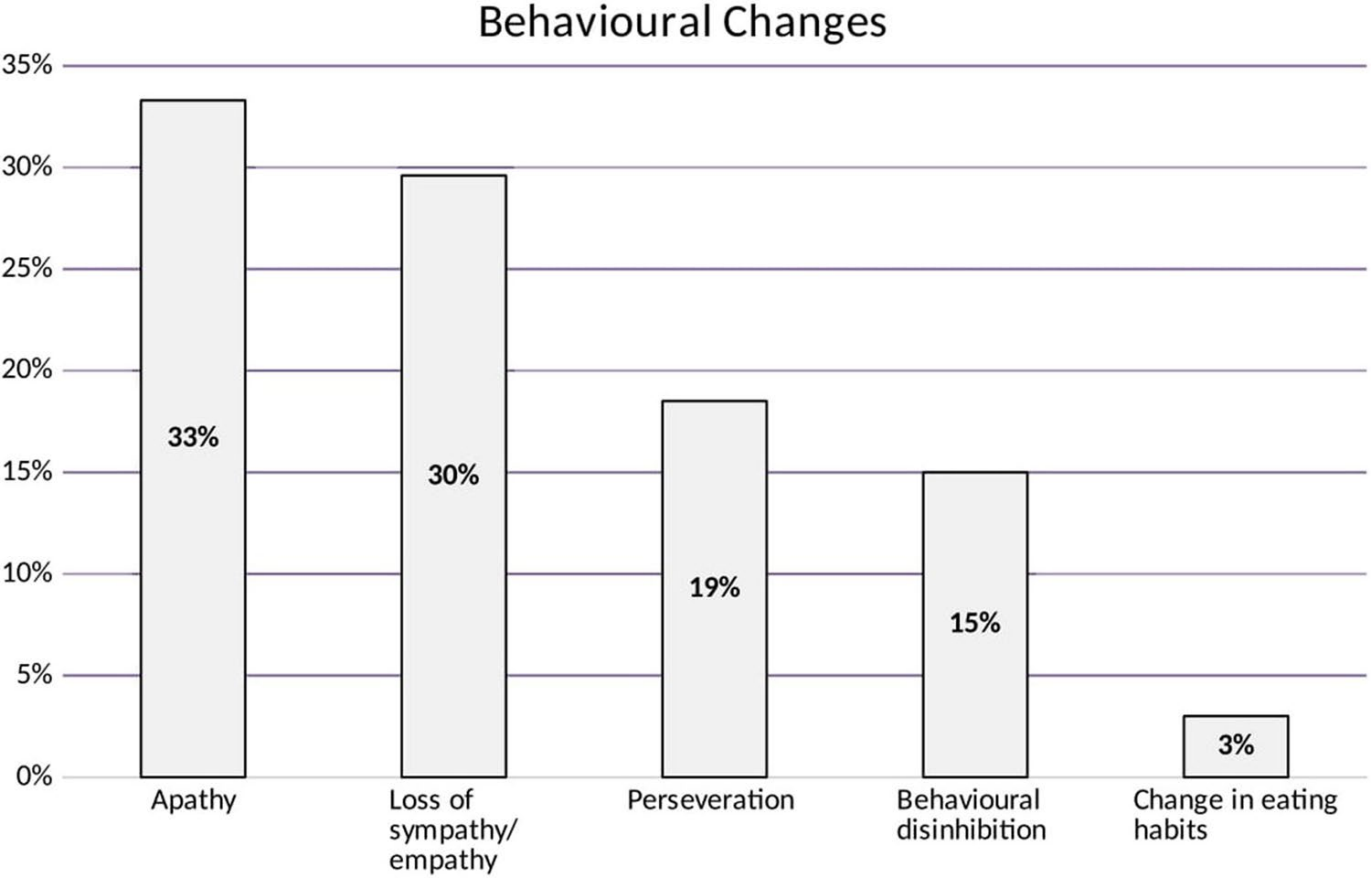
Behavioral changes in ALS patients, which shows apathy to be the most frequent one (33% of cases) and change in eating habits being the least frequent (3%)

In the behavior screen, 43% showed impairment in at least one behavioral domain. All domains were affected, most frequently apathy (33.3%) and loss of sympathy/empathy (29.6%), while changes in eating habits (3%) were the least affected (Fig. 4).

Two patients (3%) showed psychotic changes in the form of hallucinations with persecutory behavior and met the criteria for FTD.

## Discussion

The ECAS provides a reliable assessment tool of cognitive and behavioral functions for ALS; it identifies the impaired domains and their extent, which have monumental implications for the patient’s management and prognosis.

The application of the ECAS-*EG* on 62 Egyptian ALS patients showed that the test is reliable, practical, and intelligible, with inter-rater reliability and good internal consistency.

There was a need for slight adaptations to the Arabic version to make it coherent (grammatically and semantically).

Both MoCA and ECAS total scores were correlated in ALS patients. Out of 62 examined ALS patients, 39 were diagnosed to have cognitive impairment by ECAS-*EG* with an estimated cut-off value of less than 104, which is quite revealing to the extent of cognitive impairment in ALS and how far it can go unnoticed.

In our study, 63% of Egyptian ALS patients had cognitive impairment and 66% had impaired capabilities on ALS-specific tests. These results are higher than other validations, such as the recent Tunisian study with cognitive impairment in 31% total ECAS-*AR* and 32% on ALS-specific tests [18], the UK study with 29% for both scores [23], the Italian (37% and 36%), and the Chinese (35% and 42%) adaptations [12, 15].

We found that the most affected domains are executive functions and verbal fluency which is in accordance with previous UK studies [23, 24] but different from the Tunisian study which reported language impairment to be more prevalent than verbal fluency impairment [18].

The high frequency of memory impairment in our study can be attributed to the advanced disease stage [25] or to the relatively lower level of education in our cohort [26].

Our results revealed ALS-nonspecific dysfunctions in 30% of ALS patients, which is slightly higher than other studies: the Tunisian (25%) and the Italian (21%) [12, 18]. This could also be attributed to patients’ educational levels.

We found that 43% of ALS patients had a behavioral impairment, with apathy being the most prevalent impairment which is in accordance with the UK study [23, 24] and the Tunisian study (45%) [18].

## Limitations

Further research is needed to address the difficulties faced in our study, and a larger sample is needed to validate the results. Moreover, patients should be recruited from multiple centers across Egypt to allow for variability in terms of education levels, occupation, residency, and environmental factors. It would be even more beneficial to perform regional studies comparing different Arabic-speaking populations. Further investigations are required to recruit a normative sample of healthy individuals, from which demographically adjusted norms should be derived [12, 18].

We also recommend the translation of the alternative versions of the ECAS suitable for measuring change over time in ECAS versions B, C. [27]. Indeed, it will be interesting to consider the genetic mutations of the patients and their impact on the cognitive and behavioral aspects of ALS.

In conclusion, ECAS-EG was shown to be reliable and valid among Egyptian ALS patients. It provides a useful tool to monitor the cognitive and behavioral aspects of the disease in Egyptian patients in both clinical practice and scientific studies.

## Supplementary information

Below is the link to the electronic supplementary material.

Supplementary file1 (XLSX 12 KB)

Supplementary file2 (XLSX 13 KB)

## Data Availability

The data used to support the findings of this study are available within the article and its supplementary material. Raw data of the study are available from the corresponding author, upon reasonable request.
